# Mechanisms behind species-specific water economy responses to water level drawdown in peat mosses

**DOI:** 10.1093/aob/mcaa033

**Published:** 2020-03-18

**Authors:** Fia Bengtsson, Gustaf Granath, Nils Cronberg, Håkan Rydin

**Affiliations:** 1 Department of Plant Ecology and Evolution, Evolutionary Biology Centre, Uppsala University, Norbyvägen 18D, SE-752 36 Uppsala, Sweden; 2 Department of Biology, Lund University, Ecology Building, SE-22362 Lund, Sweden

**Keywords:** *Sphagnum*, bulk density, moss water content, ecohydrology, hyaline cell, leaf anatomy, pore size, water retention

## Abstract

**Background and Aims:**

The ecosystem engineers *Sphagnum* (peat mosses) are responsible for sequestering a large proportion of carbon in northern peatlands. Species may respond differently to hydrological changes, and water level changes may lead to vegetation shifts in peatlands, causing them to revert from sinks to sources of carbon. We aimed to compare species-specific responses to water level drawdown within *Sphagnum*, and investigate which traits affect water economy in this genus.

**Methods:**

In a mesocosm experiment, we investigated how water level drawdown affected water content (WC) in the photosynthetically active apex of the moss and maximum quantum yield of photosystem II (i.e. *F*_v_/*F*_m_) of 13 *Sphagnum* species. Structural traits were measured, and eight anatomical traits were quantified from scanning electron microscopy micrographs.

**Key Results:**

Mixed-effects models indicated that at high water level, large leaves were the most influential predictor of high WC, and at low water level WC was higher in species growing drier in the field, with larger hyaline cell pore sizes and total pore areas associated with higher WC. Higher stem and peat bulk density increased WC, while capitulum mass per area and numerical shoot density did not. We observed a clear positive relationship between *F*_v_/*F*_m_ and WC in wet-growing species.

**Conclusions:**

While we found that most hummock species had a relatively high water loss resistance, we propose that some species are able to maintain a high WC at drawdown by storing large amounts of water at a high water level. Our result showing that leaf traits are important warrants further research using advanced morphometric methods. As climate change may lead to more frequent droughts and thereby water level drawdowns in peatlands, a mechanistic understanding of species-specific traits and responses is crucial for predicting future changes in these systems.

## INTRODUCTION

Climate change is projected to lead to regional changes in precipitation (Meehl *et al.*, 2007). More frequent droughts and thereby water level drawdowns in peatlands (Granath *et al.*, 2016) will affect carbon storage and sequestration (Charman *et al.*, 2013) and possibly lead to vegetation shifts. In most Northern peatlands, the ground vegetation is dominated by peat mosses (*Sphagnum*) that engineer these ecosystems. To predict shifts in *Sphagnum* assemblages, we need to understand the relationships between species-specific plant traits and water economy in peatland ecosystems.

As ecosystem engineers, *Sphagnum* mosses acidify, sequester carbon and nutrients from and waterlog their surroundings, thereby forming their own habitats (Rydin and Jeglum, 2013). A thick peat layer will store water and prevent the surface moss layer from drying out, and thereby maintain moss photosynthesis and sustain growth (Waddington *et al.*, 2015). Each *Sphagnum* shoot bears a capitulum at the top, which is where the apical meristem is located and where photosynthesis occurs in the most recently formed and tightly packed branches. The optimum capitulum water content for *Sphagnum* photosynthesis, at least in measurements of detached capitula, lies between 650 and 1211 % of plant dry mass (Bengtsson *et al.*, 2016). This is high in comparison with other bryophytes (Wang and Bader, 2018). The rate of CO_2_ uptake is reduced to 50 % as water content is reduced by about 50 % from this optimum (Rydin, 1993; Schipperges and Rydin, 1998).

*Sphagnum* species have different niches related to the height above the water table (HWT) at which they grow. These niches are phylogenetically constrained, with most species of subgenus (section) *Acutifolia* growing at a higher HWT (hummock species) and most subgenus *Cuspidata* species growing at a lower HWT (hollow species) (Johnson *et al.*, 2015). The species that grow higher above the water table have adaptations to avoid and/or tolerate desiccation, while hollow species are dependent on wet periods to sustain growth (Schipperges and Rydin, 1998; Mazziotta, *et al.*, 2019). Hollow species cannot survive at higher positions, but occur scattered in hummocks together with hummock species, because they receive water from their neighbours (Rydin, 1985; Robroek *et al.*, 2007).

Although there is evidence of *Sphagnum* species being able to gradually increase tolerance to desiccation (Hájek and Vicherová, 2013), their primary strategy is to avoid desiccation so that they can continue photosynthesizing in sunny conditions without damage to the photosynthetic apparatus (Marschall and Proctor, 2004; Hájek, 2014). This is achieved with a combination of large water storage capacity, high water retention, and capillary forces that replenish the evaporating surface water from below. The large amount of small extracellular spaces in hummock species profiles is thought to be the cause of efficient capillarity and water retention in these species (Hayward and Clymo, 1982). In combination with long pendant branches that act as wicks, small pore spaces make it possible for hummock species to grow high above the water table even though they have no vascular tissue. Smaller pore sizes cause a higher bulk density (BD), i.e. more dry mass per volume (Hayward and Clymo, 1982; Golubev and Whittington, 2018). The BD of the *Sphagnum* surface layer (near-surface BD), *Sphagnum* litter (recently dead moss) and peat (more or less humified material) are considered key traits in studies about *Sphagnum* hydrology (Hayward and Clymo, 1982; Thompson and Waddington, 2008). Also, the numerical density is often described as a factor controlling the rate of water loss in *Sphagnum* since small, densely growing shoots decrease evaporation by forming a smooth surface (Elumeeva *et al.*, 2011; Laing *et al.*, 2014).

A key to the *Sphagnum* species’ ability to form and grow in peatlands is their hyaline cells, which are large, dead, tubular cells with strong walls with a capacity to hold water. It should be noted that approx. 90 % of the water content in *Sphagnum* carpets is held in extracellular pore spaces, and only around 10 % of the water is held in hyaline cells (Hayward and Clymo, 1982). However, the water inside the hyaline cells is tightly held and is the last water to be lost by evaporation. In combination, the hyaline cells and the extracellular pore spaces create a large water-holding capacity, where plant mass only constitutes 2 % of the near-surface volume (Hayward and Clymo, 1982).

In *Sphagnum* hyaline cells, water moves passively in and out through pores (Malcolm, 1996). The pores may, however, affect water retention as well as capillarity, since smaller pores reduce water loss from the cell, while they also impede rehydration of the cells (Lewis, 1988). The distribution of the pores on dorsal or ventral leaf sides renders them more or less exposed, and pores on either side could have different effects on water loss and replenishment. Similarly, the drought-sensitive chlorophyll cells can be exposed on one side more than the other. Species growing at a low HWT generally have chlorophyll cells more exposed on the dorsal side of the leaves. In the wet habitat this gives easier access of CO_2_, as diffusion is low in water. In contrast, species growing at a high HWT instead expose the chlorophyll cells on the ventral side of the leaf, which gives them more protection against desiccation (Rice and Schuepp, 1995). However, this pattern is phylogenetically confounded, and empirical evidence for this mechanism is still lacking. Furthermore, protecting the chlorophyll cells by exposing them on the inside may decrease the risk of photoinhibition, which can be tracked by measuring *F*_v_/*F*_m_, a variable known to be correlated with *Sphagnum* capitulum water content (Hájek, 2014). *F*_v_/*F*_m_ [maximum potential quantum yield of photosystem II (PSII)] is a proxy of stress on the photosynthetic apparatus, where a value below a species maximum (often between 0.7 and 0.8 in *Sphagnum* and varying among species, Korrensalo *et al.*, 2016) indicates a damaged or downregulated PSII (Murchie and Lawson, 2013).

In this study, we investigate the relationships between processes related to water economy of *Sphagnum*, and the traits potentially influencing these processes. We compare a large set of species from different habitats along the microtopographic (HWT) gradient. In a mesocosm experiment, we test how species differ in their ability to maintain capitulum water content as the water table is lowered, and how water content is related to *F*_v_/*F*_m_. First, we hypothesize that the higher above the water table a species is growing in the field, the higher is its ability to retain capitulum water when the water table is lowered. Secondly, we ask whether differences in water-holding capacity and water retention among species are best explained by anatomical traits at the micro-scale, such as leaf size and pore area of leaves, or structural features at the macro-scale, such as surface-peat BD, *Sphagnum* shoot numerical density and the area-based mass of the capitulum section (CMA). Thirdly, we ask at what capitulum water content *F*_v_/*F*_m_ starts to drop, and whether this differs between species depending on their HWT niche.

## MATERIALS AND METHODS

### Species and study sites

The field sampling was performed during the second half of August 2015, mainly at the mire complex Kulflyten in central-southern Sweden (59°54′N, 15°50′E), where the many *Sphagnum* niches make sampling of many species possible within the same ecosystem. Two species were sampled at a small rich fen, Glon, situated at the coastal land uplift area on the Swedish east coast (60°31′N, 17°55′E). The mean July and December temperatures, respectively, are 16.6 °C and –2.6 °C at Kulflyten, and 16.8 °C and –1.0 °C at Glon. Annual precipitation (1982–2013) averages 733 mm at Kulflyten and 649 mm at Glon (SMHI, 2014).

We chose 13 *Sphagnum* species representing different niches along the wetness gradient of mires and three of the larger *Sphagnum* clades (subgenera: *Acutifolia*, *Cuspidata* and *Sphagnum*). At Kulflyten we included species growing on the open bog (*S. fuscum*, *S. rubellum*, *S. balticum*, *S. tenellum*, *S. cuspidatum*, *S. majus* and *S. magellanicum*), on the pine bog (*S. magellanicum*), in the slightly minerotrophic fen soak on the bog expanse (*S. lindbergii* and *S. papillosum*), in the lagg fen on the mire margin (*S. fallax* anf *S. angustifolium*) and in the spruce swamp forest surrounding the mire (*S. girgensohnii*). In the rich fen we sampled *S. warnstorfii*, which only occurs in rich fens, and *S. fuscum* from high, probably ombrotrophic hummocks*. Sphagnum fuscum* was sampled both from the bog and from the rich fen, and we refer to them as ‘*S. fuscum* bog’ and ‘*S. fuscum* fen’. *Sphagnum magellanicum* was sampled on the open bog and pine bog, and we refer to them as ‘*S. magellanicum* open’ and ‘*S. magellanicum* pine’. Recently *S. magellanicum* was sub-divided into several species, two of which are found in the northern hemisphere: *S. medium* and *S. divinum* (Hassel *et al.*, 2018). Our *S. magellanicum* samples contain both species in both habitats (Kjell Ivar Flatberg, pers. comm.), so we treat them as *S. magellanicum sensu lato* (*s.l.*).

### Sampling and experimental set-up

Each species was sampled in 4–5 patches, which generally overlap with patches used in Bengtsson *et al.* (2016, 2018). At each patch, we carefully cut out a core from the mire surface and placed it in a PVC pipe (height = 21 cm, diameter = 16 cm).

After sampling, the cores were kept in plastic boxes in the shade of the lagg fen and supplied with water from the lagg. A week after the start of sampling, the cores were brought from the field to a climate room, where deionized water was added so that the moss capitula were 5 cm above the water table and vascular plants were cut from the surface. Temperature in the growth room was set to 15 °C and the photosynthetic photon flux density (PPFD) was 100–130 μmol m^–2^ s^–1^. The cores were sprayed with deionized water every couple of days until the start of the experiment so that the capitula would be rinsed free from residues that would otherwise accumulate in the absence of rain and cause their branch tips to blacken. The cores were randomized in the lab with three or four cores per box, but in a way that the same species would not occur twice in a box. Prior to the experiment, a few samples needed extra peat added to the base to raise them to the top of the pipe, and some were cut (from the bottom) to lower the capitula to the height of the pipe.

During sampling at Kulflyten we measured HWT at each sampling patch, while for the fen we only had HWT data from 2012. As we also had HWT data from Kulflyten 2012, we estimated the fen HWT for 2015 by assuming the same between-year difference of HWT for the fen as for Kulflyten.

### Water level drawdown experiment

The experiment was designed to test (1) the water-holding capacity of the *Sphagnum* mosses and their ability to retain water in their capitula; and (2) if water content in the capitulum is related to the stress response of PSII. The experiment started 1 week after the cores were brought to the lab. The water table was raised to 20 mm below the tips of the capitula and kept at that level for 4 d, to allow the shoots to equilibrate before measurements. The water level was then successively lowered to 50, 100, 150 and 200 mm below the capitula, and after each treatment level the mesocosms were allowed to equilibrate for 3 d before subsequent measurements. The water level was then lowered to the next level on the fourth day.

After equilibration with the water level, the lights were turned off, and after 30 min of dark acclimatisation we measured fluorescence yield (maximum quantum yield of PSII, *F*_v_/*F*_m_; see Granath *et al.*, 2009 for details) with a pulse-modulated fluorometer under very weak light conditions holding the fibre optic cable steady 11 mm from the top of a capitulum (Mini-PAM photosynthesis yield analyser; Walz, Germany). Five capitula per sample had been individually marked with plastic toothpicks, and these were used for fluorescence yield measurements throughout the experiment. The measurements were then averaged per core for each water level.

The capitulum water content measurements were destructive: three samples per core were collected, and their positions were marked to avoid later samples being a neighbouring shoot. Each sample was immediately put in a small plastic water-tight vial of known weight. The vial was weighed with the sample and the sample was then dried (65 °C) and weighed, and water content (g g^–1^ dry moss) was calculated.

### Structural measurements

Once the experiments were finished, the peat cores were drained and dried until no further weight loss was detected (65 °C). We measured numerical density (ND; number of capitula per cm^2^) using a circle with a diameter of 4 cm, and calculated the mean from four different measurements on each core. The capitulum layer (top 1.5–2 cm) was cut off and weighed to give the area-based mass of the capitulum layer (g cm^–2^; referred to as capitulum mass area, CMA), which is the same variable as ‘capitulum biomass’ in Laing *et al.* (2014) and similar to ‘canopy mass area’ used for non-*Sphagnum* mosses by Waite and Sack (2010). Additionally, bulk density (g cm^–3^) was measured in three cut out and dried sections: 5 cm immediately below the capitula (BD1), the next 5 cm below this (BD2) and the remaining core below (BD3).

### Leaf trait measurements

We measured branch leaf traits from scanning electron microscopy (SEM) micrographs using the software ImageJ (Fiji; Schindelin *et al.*, 2012). To obtain the images, we used moss material from one sample per species, collected in 2013 at the same patches as the experimental material. We used branches just beneath the capitulum of a shoot, and picked out leaves from the middle of a normal looking spreading branch. The moss leaves were mounted on aluminium stubs for SEM using glue – no other preparation of the material was performed – and images were acquired under 5000 V in the scanning electron microscope (Hitachi SU3500).

**Fig. 1.**
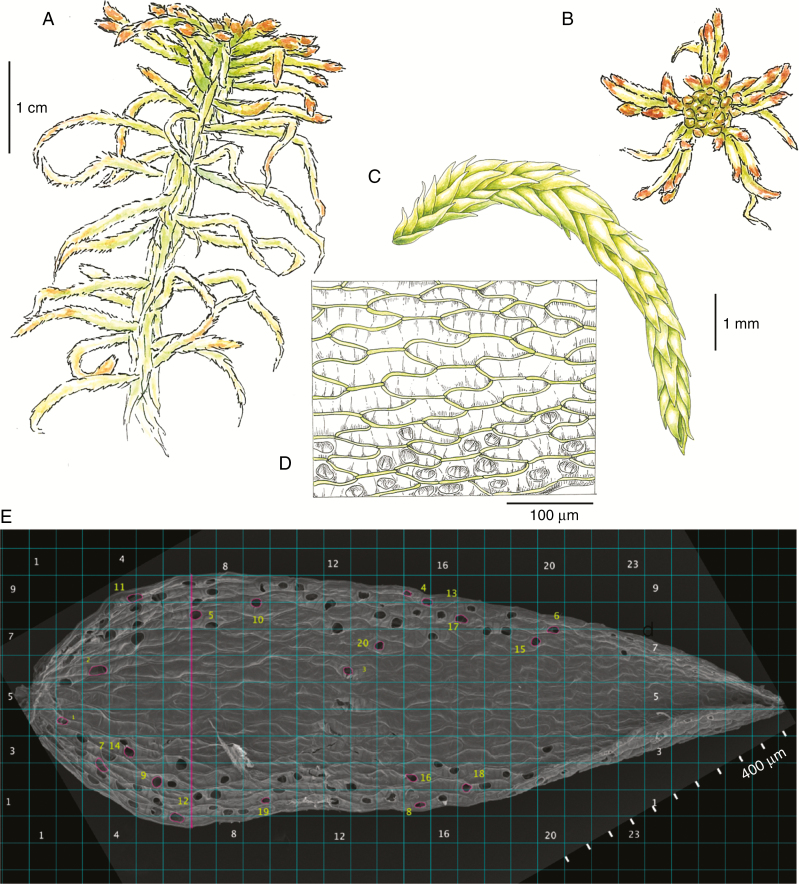
(A) A *Sphagnum* shoot with a capitulum at the top; side view. (B) Capitulum with apical meristem and tightly packed branches; view from above. (C) Branch with overlapping leaves (A–C, *S. fallax*). (D) Leaf, one cell layer thick, constituting hyaline and chlorophyll cells (*S. fuscum*; ventral side). (E) Leaf measurements in ImageJ illustrated by a ventral leaf side of *S. fuscum*. Leaf length and width were measured (mm). Along the widest section of the leaf (purple vertical line). the widths of exposed chlorophyll cells were measured. A grid was set up (teal) and grid squares were randomized to choose 20 pores (yellow numbers). Areas (µm^2^; purple) and diameters of pores along the leaf length were measured. Illustrations: Fia Bengtsson.

For each sample, we measured morphological traits on two leaves of which one showed the dorsal side and one showed the ventral side. Leaf length and width were estimated as the average from the two leaves (µm). On each leaf surface, 20 pores were randomly chosen and the area (µm^2^) and diameter (µm) in the longest direction of each of these pores were measured (Fig. 1). The total number of pores on the leaf was counted, and the pore area was calculated as a percentage of the leaf area measured using the software ImageJ. Chlorophyll cell exposure (proportion of leaf area) was measured along the widest section of the leaves, and calculated as the summed widths of the exposed chlorophyll cells divided by the total leaf width. If parts of the section were not clearly hyaline or chlorophyll cells, they were excluded (Fig. 1).

### Statistical analysis

We used a mixed-effects model to estimate species-specific responses in capitulum water content (WC) to water level drawdown, with an interaction specified for the fixed factors water level (WL) and species. Sample (i.e. core) ID was included as a random factor to account for repeated sampling and specified as varying intercept and slope. We extracted three WC responses to WL below the moss surface for each sample: WC at WL 20 mm (WC_20_), WC at WL 200 mm (WC_200_) and the slope of the regression lines between WC and WL during the lowering of the water level from 20 to 200 mm (WC_slope_, extracted from the mixed-effect model for each sample).

To quantify the influence of morphological and structural variables on WC_slope_, WC_20_ and WC_200_, we fitted linear mixed models (LMMs) with species as a random factor. Bulk density and structural traits (e.g. ND) were recorded for all samples, while data on leaf anatomical variables were scored only at the species level (or species–environment combinations, e.g. *S. magellanicum*). Hence, we had predictors on both the sample and group level in our models, and many predictors were strongly intercorrelated. To test these predictors and explore possible alternative models, while considering the sample sizes and intercorrelations, we used a three-stage modelling approach where predictors were included based on the research questions and what we know about *Sphagnum* biology. First we defined the overall model structure specification. We avoided strong collinearity by only allowing either ND or CMA (sample-level variables), either leaf length or width, and only one anatomical character out of six in a model. For the response WC_20_, only the top bulk density section (BD1) was included since the lower sections were completely water saturated during this part of the experiment. Secondly, we identified four models per response variable to test the predictors we expected to be important based on (eco-)hydrological models (BD, ND or CMA) or earlier literature (leaf width, and leaf dorsal pore size or area as they are more exposed to evaporation compared with the ventral side). Leaf width, rather than leaf length, was chosen because a wider leaf makes it possible to hold more water by enabling a more curved leaf. Dorsal chlorophyll exposure was strongly correlated with pore area/size (*r* > 0.7), which are variables directly related to water storage (Hájek and Beckett, 2008; McCarter and Price, 2014), while chlorophyll exposure is not expected to affect water content (Hájek and Beckett, 2008). We therefore decided to exclude these variables from the models, but they are included in the publicly available data set for future analyses. Thirdly, we ran all possible model combinations, given the defined model structure above, and ranked the models based on the AICc (Akaike information crtiterion corrected for sample size). The two models with the lowest AICc were included as best predicting models among our set of models (Burnham and Anderson, 2004). All models within four AICc units from the lowest AICc model can be found in Supplementary data Table S1.

To investigate the relationship between *F*_v_/*F*_m_ and water content for each species, we largely followed the approach described by Hájek and Beckett (2008) and fitted an asymptotic function {*F*_v_/*F*_m_ = *a *− (*a *− *b*) × exp[−log_e_(*c*)] × WC} to the data. Here, WC is the measured water content, *a* is the asymptotic maximum *F*_v_/*F*_m_ value, *b* is the intercept and *c* describes the relative increase in *F*_v_/*F*_m_ while WC is increasing. This function is a slightly different asymptotic function from that which Hájek and Beckett (2008) used but it fitted our data better and converges more easily. The function was fitted as a non-linear model using generalized least squares where a compound symmetry correlation structure was applied to account for within-sample dependence. The point when a lower water content in the capitula results in a decline of *F*_v_/*F*_m_ was defined as 0.95*a* (Hájek and Beckett, 2008). We used this point estimate of water content as a way to rank the species along the tissue water content variable to indicate in which order they are affected by losing water, rather than claiming that this is a stress threshold for each species.

All analyses were performed in the statistical software R v.3.4.0 (R Core Team, 2017), and LMMs were fitted using the lme4 package (version 1.1-17; Bates *et al.*, 2015). Classic residual checks were performed, and the response WC_slope_ was multiplied by –1 and log_e_ transformed to produce a homogenous error distribution. For the LMMs, *R*^2^ values (conditional and marginal) and AICc values were extracted using the package MuMIn (version 1.42.1, Bartoń, 2018). For AICc values, we fitted the models with maximum likelihood, while restricted maximum likelihood (REML) was used to extract coefficient estimates and *R*^2^ values. The non-linear models were fitted using the gnls function in the nlme package (version 3.1-142, Pinheiro *et al.*, 2019), and a pseudo-*R*^2^ was calculated based on the residuals (Lefcheck, 2016): *σ*_fitted_/(*σ*_fitted_ + *σ*_residuals_). We used the function Anova in the package car to produce *P*-values for fixed factors (version 3.0-0, Fox and Weisberg, 2011). Principal component analysis (PCA) of the leaf traits of the species was performed using the function prcomp in base R. Data is available from the Figshare repository, doi: 10.6084/m9.figshare.12034353.

## RESULTS

### Water content at water level drawdown

**Fig. 2.**
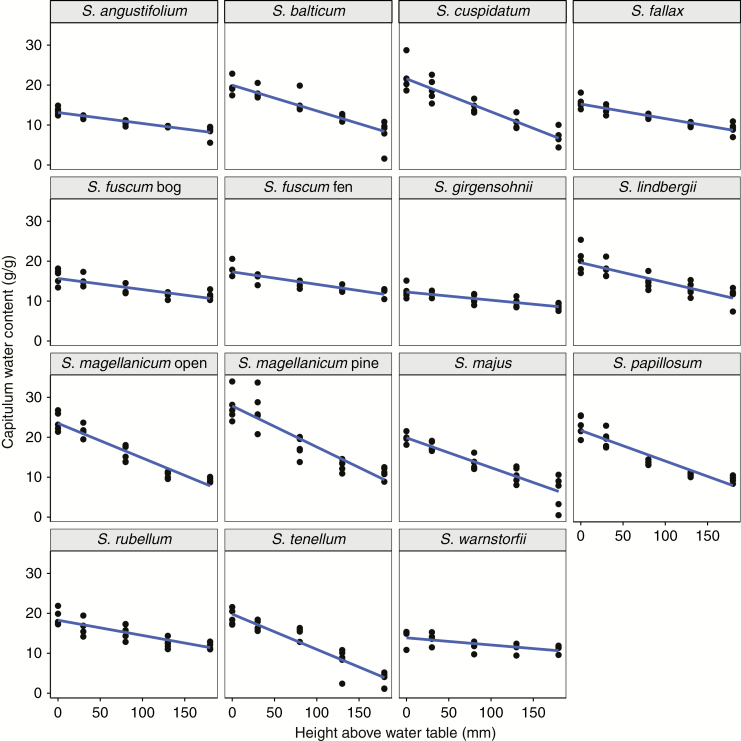
Capitulum water content measured in samples of 13 *Sphagnum* species as the water table was lowered from 20 to 200 mm below the surface. Lines illustrate the linear relationship between capitulum water content and water level; the slope of the line defines the parameter WC_slope_ (g g^–1^ mm^–1^) at the species level; *n* = 4–5 per species. Note that in subsequent statistical analyses, individual values of WC_slope_ were used for each sample. See Fig. 3 for the values of WC_slope_.

The water content measurements at 20 and 200 mm were uncorrelated (*r *= 0.11 for WC_20_ vs. WC_200_; d.f. = 71, *P* = 0.4). We used the slope of the regression for each species between water content (WC) and water level (WL) (WC_slope_; Fig. 2) as an integrated response to changes in water level. By statistical necessity WC_slope_ is correlated with the other measures; more strongly so for WC_20_ (*r* = –0.84, *P* < 0.0001) than for WC_200_ (*r* = 0.43, *P* < 0.001).

**Fig. 3.**
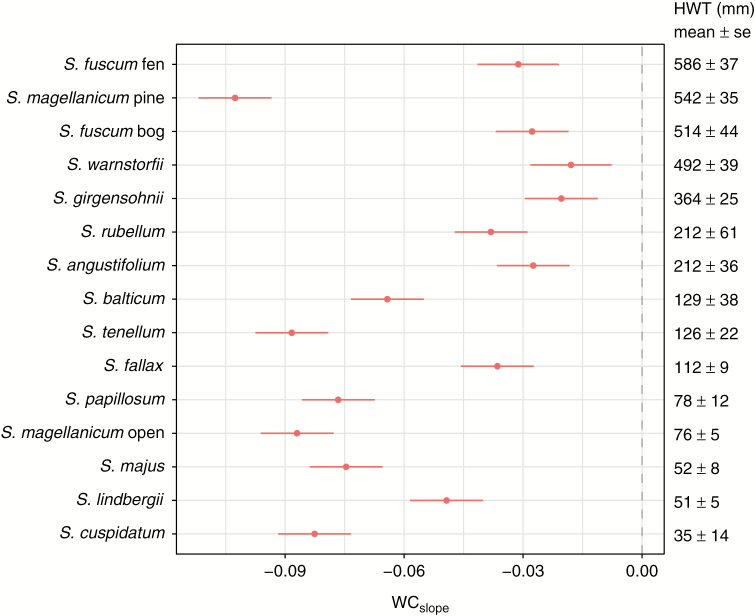
The slopes (g g^–1^ mm^–1^) of the regression lines between capitulum water content and water level (WC_slope_; Fig. 2) in the water level drawdown experiment plotted with mean and 95 % confidence interval for each species. The species are ranked according to their mean height above the water table (HWT; shown on the right side of the figure). WC_slope_ is based on five repeated measurements of 4–5 cores per species (*n* = 365).

Our fitted LMM identified differences between species in WC_slope_ (species × WL interaction: *F*_15,277_ = 171.4, *P* < 0.0001). This model explained 91 % (*R*^2^_conditional_) of the total variation in capitulum WC, while species and WL together explained 84 % of the total variation (*R*^2^_marginal_), and WL alone explained 54 %. Ranking species’ WC response to WL (i.e. WC_slope_) according to their HWT positions in the field, from driest (hummock) to wettest (hollow), revealed that the lawn-hollow species had steeper slopes than the hummock species (Fig. 3). There were a few exceptions though: the hollow-dwelling *S. fallax* and *S. lindbergii* had slopes more similar to the hummock species, and one of the driest growing species, *S. magellanicum* pine had the steepest negative slope of all species.

### The effects of leaf traits and structural traits on water holding and water retention

We summarize the ranges and means of the measured structural and anatomical traits in Supplementary data Table S2. From our pre-defined models for each response, we found that, overall, the results were fairly robust to model specification (Table 1). Our pre-defined models (i.e. hypothesized models) predicted the responses WC_20_ and WC_slope_ better than they did WC_200_, and these models were similar to the models ranked highest according to AICc values (Table 1). For WC_20_, the predictors in our four pre-defined models explained 50–55 % of total variation, for WC_slope_ 51–66 % and for WC_200_ 11–16 %.

**Table 1.** Mixed-effects models of the responses WC_20_, WC_200_ and WC_slope_, including as predictors: (1) one of the anatomical leaf traits (*n* = 15); (2) either leaf length or width (*n* = 15); (3) either capitulum mass per area (CMA) or shoot numerical density (ND) (*n* = 73); and (4) one or more of the bulk density (BD) sections (*n* = 73)

Models	Intercept	Dorsal pore area (%)	Dorsal pore size (µm)	Dorsal pore number	Ventral pore area (%)	Ventral pore size (µm)	Ventral pore number	Leaf length	Leaf width (mm)	CMA (g cm^–2^)	ND (cm^–2^)	BD1 (g cm^–3^)	BD2 (g cm^–3^)	BD3 (g cm^–3^)	*R* ^2^m/ *R*^2^c	d.f.	AICc
Response: WC_20_																	
Rank 1 AICc	12.25 ± 1.68	–0.12 ± 0.06							11.79 ± 2.13			107.5 ± 37.5			55/74	6	363.2
Rank 2 AICc	14.90 ± 2.22				–0.38 ± 0.17				8.69 ± 2.38		–0.37 ± 0.21	118.7 ± 40.2			57/75	7	363.3
Model 1	12.03 ± 1.84	–0.11 ± 0.06							11.56 ± 2.26	0.12 ± 0.35		108.1 ± 38.0			55/74	7	365.6
Model 2	12.73 ± 1.77	–0.11 ± 0.06							11.22 ± 2.24		–0.19 ± 0.21	120.8 ± 40.5			55/74	7	364.6
Model 3	12.57 ± 2.27		–0.19 ± 0.15						12.51 ± 2.62	0.05 ± 0.37		112.1 ± 38.6			51/74	7	367.9
Model 4	12.90 ± 2.07		–0.16 ± 0.15						11.97 ± 2.67		–0.15 ± 0.23	121.2 ± 41.5			50/74	7	367.4
Response: log_e_WC_slope_																	
Rank 1 AICc	–3.26 ± 0.19	–0.02 ± 0.01							1.25 ± 0.27				10.2 ± 2.5	–8.6 ± 2.2	64/87	7	25.2
Rank 2 AICc	–2.85 ± 0.27					–0.07 ± 0.02			1.38 ± 0.29				10.3 ± 2.5	–8.8 ± 2.2	62/87	7	26.3
Model 1	–3.34 ± 0.22	–0.02 ± 0.01							1.29 ± 0.28	–0.01 ± 0.04		3.6 ± 4.6	9.2 ± 3.0	–8.2 ± 2.3	66/87	9	29.3
Model 2	–3.33 ± 0.23	–0.02 ± 0.01							1.27 ± 0.28		–0.003 ± 0.024	3.8 ± 4.8	9.0 ± 2.9	–8.1 ± 2.3	65/87	9	29.4
Model 3	–3.20 ± 0.29		–0.04 ± 0.02						1.46 ± 0.36	–0.01 ± 0.04		3.2 ± 4.7	9.5 ± 3.0	–8.5 ± 2.3	52/86	9	35.7
Model 4	–3.22 ± 0.29		–0.04 ± 0.02						1.44 ± 0.37		0.0004 ± 0.03	3.2 ± 4.9	9.3 ± 3.0	–8.5 ± 2.3	51/86	9	35.8
Response: WC_200_																	
Rank 1 AICc	3.69 ± 1.92					0.32 ± 0.15								33.9 ± 13.9	19/64	5	336.1
Rank 2 AICc	6.16 ± 1.17	0.10 ± 0.06												33.6 ± 13.9	17/64	5	337.2
Model 1	5.47 ± 1.91	0.11 ± 0.06							0.92 ± 2.40	–0.01 ± 0.32		12.5 ± 40.5	1.4 ± 26.5	32.8 ± 20.2	16/66	9	346.9
Model 2	5.27 ± 2.00	0.11 ± 0.06							1.05 ± 2.46		0.05 ± 0.21	10.7 ± 42.0	1.7 ± 26.0	32.8 ± 20.1	16/68	9	346.9
Model 3	4.61 ± 2.27		0.21 ± 0.15						–0.15 ± 2.65	0.05 ± 0.33		11.4 ± 40.8	–2.3 ± 26.3	36.0 ± 20.1	11/66	9	348.0
Model 4	4.70 ± 2.23		0.21 ± 0.16						–0.07 ± 2.74		–0.01 ± 0.22	11.7 ± 42.3	–1.6 ± 25.9	36.1 ± 20.0	11/66	9	348.0

Each model estimate is shown as ± s.e. Species is included as a random factor (*n* = 15). The models are arranged under each response starting with the models resulting with the lowest AIC, and then our original/hypothesized models (see the Materials and Methods). Marginal and conditional *R*^2^s (*R*^2^m, *R*^2^c) show the amount of the total variance that is explained by predictors, and predictors and species, respectively. AICc is a measure of model fit where the lowest value indicates the best relative fit

The canopy traits (ND and CMA) were not important predictors in our models, and the coefficients showed large standard errors (Table 1). In contrast, leaf width was consistently important, except for WC_200_. Pore area (dorsal or ventral) was also a meaningful variable in our models but could be interchanged by ventral pore size for WC_slope_ and WC_200_.

The bulk density variables, which were measured at different depths of the cores, showed strong effects on all WC responses (Table 1). For WC_slope_, the two lower BD sections had larger effects in the pre-defined models than the top section, and the top section was not included in the highest ranked AICc models. For WC_200_, only BD3 was included in the top AICc models.

### The relationship between *F*_v_/*F*_m_ and capitulum WC

**Fig. 4.**
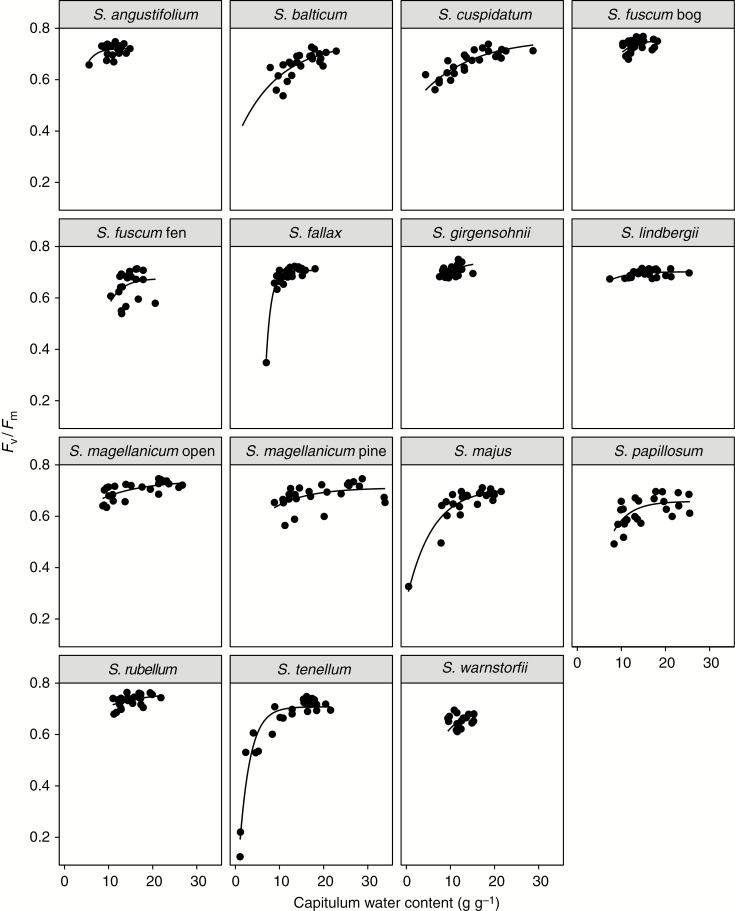
Capitulum water content as a predictor of chlorophyll fluorescence (*F*_v_/*F*_m_) under a water table drawdown. The lines show the fitted asymptotic regression line for each species based on five repeated measurements of 4–5 samples (*n* = 20–25 per panel).

**Fig. 5.**
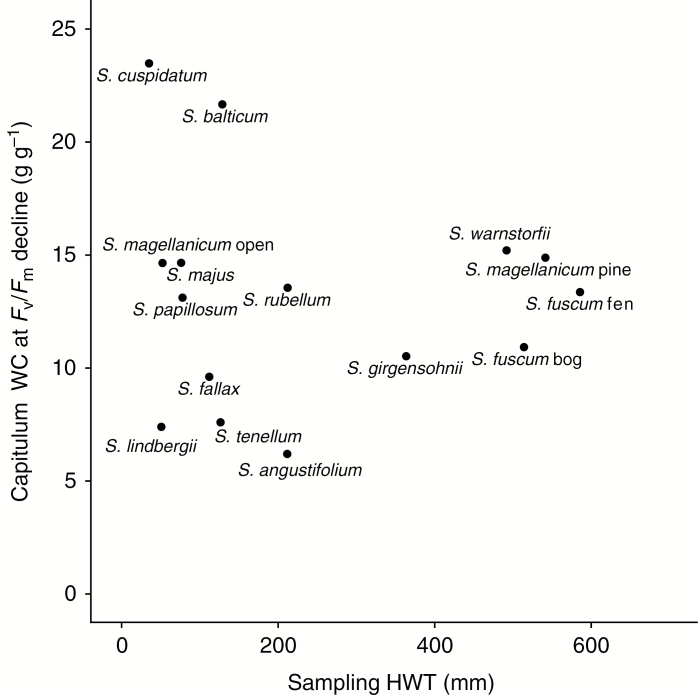
The capitulum water content when *F*_v_/*F*_m_ starts to decline during desiccation, plotted against mean sampled HWT in the field for each species.

Species-specific asymptotic regression models showed an overall good fit of the relationship between *F*_v_/*F*_m_ and capitulum WC (Fig. 4). The species that had a poor fit, i.e. low pseudo-*R*^2^, were species that lost little water during the experiment (mainly the hummock species). Their narrow range in WC made it difficult to fit a model that explains much of the variation. In contrast, some hollow species with a larger WC range showed good model fit, e.g. *S. tenellum* (pseudo-*R*^2^ = 92 %), *S. majus* (pseudo-*R*^2^ = 85 %) and *S. cuspidatum* (pseudo-*R*^2^ = 74 %) (Supplementary data Table S3). From these models, we calculated at what WC a decline of *F*_v_/*F*_m_ occurred for each species. We found no clear relationship with HWT among species, but the variation among wet-dwelling species was higher than among the dry-dwelling species (Fig. 5).

## DISCUSSION

### WC at water level drawdown and adaptations to different HWT niches

We interpret our three water content responses to represent maximum water-holding capacity (WC_20_), water retention capacity (WC_slope_) and desiccation avoidance (WC_200_).

We expected hummock species to show desiccation avoidance during the water table drawdown as this prolongs the period in which they can continue to grow during dry spells. In line with our prediction, most species in our experiment that were sampled high above the water table retained water during drawdown, leading to high WC_200_, which fits the theory of hummock species avoiding desiccation (Marschall and Proctor, 2004; Hájek, 2014). Also, species with a higher maximum water-holding capacity lost more water during the WT drawdown, and consequently exhibited a similarly low WC_200_ to the other species. However, there was one exception to the hollow–hummock pattern with slower water loss in hummock species; the hummock-growing *S. magellanicum* pine had a steep WC_slope_. We interpret this as an alternative strategy for a hummock species: to avoid desiccation by having a higher maximum water-holding capacity, rather than losing water at a slow pace. To our knowledge, there are no prior observations of this alternative strategy of hummock species for survival high above the water table.

Elumeeva *et al.* (2011) found that although colonies of the hummock species *S. fuscum* lost water more slowly than hollow species, the evaporation rate was on a par with that of the hollow species *S. lindbergii*. We also found that *S. fuscum* behaves as expected for a hummock species, and that *S. lindbergii* behaved more like a hummock species than a hollow species in terms of water loss. Similarly, the hollow-dwelling species *S. fallax* behaved differently from other hollow species as it lost water to a similar degree as hummock species. If these water economy traits are important in the field, our finding should not be surprising as *S. fallax* and *S. angustifolium*, which is a hummock-growing species in the same part of the mire as *S. fallax*, are closely related and supports the hypothesis of phylogenetic constraint on niche evolution in *Sphagnum* (Johnson *et al.*, 2015).

### Factors affecting water holding and retention

We found that leaf width was among the most important traits increasing maximum water-holding capacity and water retention, as most models supported this trait as a predictor. For WC_20_ and WC_slope_, leaf width was included in the models with the lowest AIC, while it was excluded from the top WC_200_ models. Larger leaves can be associated with larger holding capacity, suggesting greater pore space between the leaves. As a comparison, Såstad and Flatberg (1993) found evidence of shorter leaves in drier conditions within *S. strictum*, and that more curved leaves retained water more efficiently (i.e. the convexity of leaves, or how cucullate they are), and they argued that curvature is more likely to be the trait with most influence on water economy than leaf area.

Leaf curvature directly influences water-holding capacity by creating larger pore spaces that do not drain easily (Malcolm, 1996). The subgenus *Sphagnum* has ‘hooded’ and strongly curved leaves that provide higher water holding-capacity. In the species we sampled, curvature and leaf size are strongly related. *Sphagnum magellanicum* pine had the greatest water-holding capacity and the fastest water loss, indicating a large pore space but mainly consisting of larger spaces that drain rapidly during water level drawdown (McCarter and Price, 2014). This species had the second driest realized niche at our field sites (high hummocks in the pine bog), while *S. magellanicum* open grew closer to the water table on the open bog. This intraspecific variation does not seem to exactly fit the recent division of *S. magellanicum* into two species in Europe (Hassel *et al.*, 2018), since both *S. divinum* and *S. medium* appeared in both habitats.

We speculate that hummock formation for this type of species in the pine bog without the high bulk density usually associated with hummock species is possible through leaf trait-driven high maximum water-holding capacity. In the pine bog, evaporation is lower as the tree canopy reduces incoming radiation and wind (Waddington *et al.*, 2015). This makes it possible for *S. magellanicum* pine to take advantage of rain events through a large storage capacity, and, because competition for light is more important under a canopy, looser colonies and larger shoots are beneficial (Mazziotta *et al.*, 2019). In open habitats, higher BD and a smoother moss surface are necessary, because higher BD results in stronger capillary forces and a smoother canopy hampers evaporation (Elumeeva *et al.*, 2011; Laing *et al.*, 2014; Waddington *et al.*, 2015). The anatomical traits related to hyaline pore size and area were important for predicting water content and retention in both pre-defined models and those selected by AICc.

At lower water tables (lower water potential), the extracellular water pool will drain fast and the proportion of smaller pore spaces and their connectivity becomes important for the maintenance of water in the capitula (McCarter and Price, 2014). This occurs around a WC of roughly 1000 % (Hayward and Clymo, 1982; Rezanezhad *et al.*, 2016), which suggests that our drawdown had less impact on intracellular water storage (here we include hyaline cells) as many species’ water content did not decrease far below 1000 %. Hyaline cell pore size and area, and chlorophyll cell placement have previously been discussed to affect the water loss of the hyaline cells (Lewis, 1988), but otherwise the literature on the adaptive value of pore area is scarce. Li *et al.* (1992) found a greater number of pores in dry conditions (within species). However, we did not detect any influence of pore numbers on water storage/retention, indicating that pore size/area should be further studied. In our models we did not include chlorophyll cell placement as it was strongly correlated with pore-related variables, but this is also a variable to explore in future studies. In addition, our PCA (Supplementary data Fig. S1) indicates that these traits aggregate at subgenus level, where small pore size and pore area and exposure of chlorophyll cells to the dorsal side are associated with species belonging to subgenus *Cuspidata*. Thus, it remains to be determined to what extent these leaf traits are mechanistically important for the water economy of *Sphagnum*, the plasticity in these trait values and the potential covariation with phylogeny.

Higher near-surface BD should increase capillarity and water retention, and thereby maintain a high capitulum WC during a water table drawdown. The top BD section (2–7 cm) strongly influenced water content at 2 cm water level, but was not important for predicting the water retention and content under water level drawdown. This is somewhat expected as the lower sections will determine the conductivity of the peat water column (smaller pore sizes means greater capillarity), thereby upholding capitulum WC in the event of drought (Hayward and Clymo, 1982). More surprising was the weak influence of ND on WC. However, this may be a consequence of minimal air movement in the lab environment, while in the field ND increases water-holding capacity by decreasing the evaporation through increased surface evenness (Elumeeva *et al.*, 2011; Waddington *et al.*, 2015).

Geometric morphometrics are still not widely used for research on bryophyte traits although there are many potential applications (Stanton and Reeb, 2016). For example, measuring traits on the convex *Sphagnum* leaves could be done more accurately using 3-D image processing. More advanced morphometric analyses could, in addition to more accurate measurements of anatomical traits, be used to estimate the intra- and extracellular holding capacity of a *Sphagnum* leaf (Hájek and Beckett, 2008). In addition to the anatomical traits, traits at the shoot level, such as branch lengths and fascicle density along the stem, contribute to water economy traits (Hayward and Clymo, 1982; Såstad *et al.*, 1999). Morphometric analyses could also be used to estimate traits at the canopy scale, as has been done using laser scanning microscopy (Rice *et al.*, 2005). Traits at all these levels have bearing on the adaptations to different heights above the water table.

### Changes in chlorophyll fluorescence related to water content

Being able to sustain photosynthesis at lower water content and the ability to restore photosynthesis after severe droughts are manifestations of desiccation tolerance. Here, we used *F*_v_/*F*_m_ as an indicator of water stress in *Sphagnum* as chlorophyll fluorescence has been used as a functional trait in *Sphagnum* before, for example in Laine *et al.* (2011), Kangas *et al.* (2014) and Korrensalo *et al.* (2016).

*F*_v_/*F*_m_ could be modelled from water content quite well in hollow species, while in hummock species the water table drawdown did not affect water content enough to cause substantial effects on *F*_v_/*F*_m_ (Fig. 4). These results support the idea of hummock species not only resisting desiccation, but also tolerating it. These stabilizing processes result in high variation in the *F*_v_/*F*_m_ response to water table drawdown among species growing high above the water table, and high among species growing closer to the water table (Fig. 5). The environment on hummocks is very constrained, which limits how species can respond to change, whereas in hollows species multiple strategies are possible. However, it should be noted that we performed a short-term experiment and the capitulum water content may need to be low for a longer time to damage PSII. Some mosses, particularly the hummock species, may also have ‘hardened’ and developed desiccation tolerance (Hájek and Vicherová, 2013) during the slow desiccation in these species. This may have contributed to the relatively small decreases in chlorophyll fluorescence that we found.

### Conclusions

We found overall support for the hypothesis that there is a negative correlation between HWT position in the field and capitulum water retention under water table drawdown. Furthermore, we suggest that higher water content under drought can be achieved either by slow water loss or by a high maximum water-holding capacity. We found that chlorophyll fluorescence is linked to water content, but in our models this relationship was only strong for hollow species.

We found some evidence that anatomical traits explain the water economy responses. In particular, leaf width was important for maximum water-holding capacity and water retention, which we attributed to leaf convexity and large extracellular spaces. Cell-level traits linked to hyaline pore size and pore area also appeared to influence water-holding capacity and retention. However, leaf and cell trait variation was largely correlated with species identity, which limits mechanistic inference, and further studies are needed to test our results. Bulk density largely controlled the water retention curve and capitulum water content, but bulk density at different depths affected different responses. We conclude that quantification of anatomical traits with advanced morphometric methods is worth pursuing to improve our mechanistic understanding of the ecohydrology of *Sphagnum*-dominated peatlands.

## SUPPLEMENTARY DATA

Supplementary data are available online at https://academic.oup.com/aob and consist of the following: Table S1: mixed-effects models of the responses WC_20_, WC_200_ and WC_slope_. Table S2: the ranges and means of the traits measured for 13 *Sphagnum* species. Table S3: species-specific asymptotic regression models were fitted for the relationship between *F*_v_/*F*_m_ and water content. Figure S1: PCA of the leaf traits of the *Sphagnum* species.

mcaa033_suppl_Supplementary_Material

## FUNDING

This study was supported by the Swedish Research Council (contract 2015-05174 to H.R.) and by a scholarship from The Royal Swedish Academy of Sciences to F.B.
