# Supplementary material for: Mechanisms behind species-specific water economy responses to water level drawdown in peat mosses
Source: Ann Bot. 2020 Mar 18;126(2):219–30. doi: 10.1093/aob/mcaa033 (PMC7523593; doi:10.1093/aob/mcaa033)
Supplement: mcaa033_suppl_Supplementary_Material [file mcaa033_suppl_supplementary_material.docx]

## Supplementary material

| **Supplementary material** Table S1. Mixed effects models of the responses WC20, WC200 and WCslope, including as predictors 1) one of the anatomical leaf traits (n=15), 2) either leaf length or width (n=15), 3), either capitulum mass per area (CMA) or shoot numerical density (ND) (n=73), and 4) one or more of the bulk density (BD) sections (n=73). Species is included as a random factor (n=15). AICc is a measure of model fit where the lowest value indicates the best relative fit, here the models for each response with AICc within 4 units from the model with the lowest AICc are shown. Marginal and conditional R^2^s (R^2^m, R^2^c) show the amount of the total variance that is explained by predictors, and predictors and species, respectively. | | | | | | | | | | | | | | | | |
| --- | --- | --- | --- | --- | --- | --- | --- | --- | --- | --- | --- | --- | --- | --- | --- | --- |
| Intercept | Dorsal pore area (%) | Dorsal pore size (µm) | Dorsal pore number | Ventral pore area (%) | Ventral pore size (µm) | Ventral pore number | Leaf length (mm) | Leaf width (mm) | CMA (g cm^-2^) | ND (cm^-2^) | BD1 (g cm^-3^) | BD2 (g cm^-3^) | BD3 (g cm^-3^) | R^2^m/ R^2^c | df | AICc |
| Response: WC_20_ | |  |  |  |  |  |  |  |  |  |  |  |  |  |  |  |
| 12.25 | -0.12 |  |  |  |  |  |  | 11.790 |  |  | 107.5 |  |  | 55/74 | 6 | 363.2 |
| 14.90 |  |  |  | -0.38 |  |  |  | 8.687 |  | -0.37 | 118.7 |  |  | 57/75 | 7 | 363.3 |
| 13.31 |  |  |  | -0.30 |  |  |  | 10.087 |  |  | 96.7 |  |  | 54/75 | 6 | 364.5 |
| 12.73 | -0.11 |  |  |  |  |  |  | 11.220 |  | -0.19 | 120.8 |  |  | 55/74 | 7 | 364.6 |
| 13.73 |  |  |  |  | -0.26 |  |  | 12.267 |  |  | 108.2 |  |  | 52/74 | 6 | 364.9 |
| 13.02 |  |  |  | -0.38 |  |  |  | 8.933 | 0.45 |  | 96.4 |  |  | 55/74 | 7 | 365.1 |
| 12.91 | -0.13 |  | 0.0009 |  |  |  |  | 9.473 |  |  | 108.7 |  |  | 55/75 | 7 | 365.1 |
| 11.08 |  |  |  |  |  |  |  | 11.479 |  |  | 114.0 |  |  | 48/74 | 5 | 365.4 |
| 12.70 |  | -0.20 |  |  |  |  |  | 12.645 |  |  | 112.0 |  |  | 51/74 | 6 | 365.5 |
| 14.54 |  |  |  | -0.40 |  |  |  | 8.388 | 0.19 | -0.31 | 115.4 |  |  | 57/74 | 8 | 365.5 |
| 12.03 | -0.11 |  |  |  |  |  |  | 11.565 | 0.12 |  | 108.1 |  |  | 55/74 | 7 | 365.6 |
| 13.53 | -0.13 |  | 0.0010 |  |  |  |  | 8.514 |  | -0.21 | 123.2 |  |  | 54/75 | 8 | 366.4 |
| 11.81 |  |  |  |  |  |  |  | 10.767 |  | -0.24 | 129.1 |  |  | 48/73 | 6 | 366.4 |
| 14.04 |  |  |  |  | -0.24 |  |  | 11.631 |  | -0.19 | 120.4 |  |  | 52/74 | 7 | 366.5 |
| 12.89 | -0.11 |  |  |  |  |  |  | 11.281 | -0.06 | -0.21 | 121.8 |  |  | 55/74 | 8 | 367.1 |
|  |  |  |  |  |  |  |  |  |  |  |  |  |  |  |  |  |
| Response: log_e_WC_slope_ | |  |  |  |  |  |  |  |  |  |  |  |  |  |  |  |
| -3.26 | -0.02 |  |  |  |  |  |  | 1.25 |  |  |  | 10.2 | -8.6 | 64/87 | 7 | 25.2 |
| -2.85 |  |  |  |  | -0.07 |  |  | 1.38 |  |  |  | 10.3 | -8.8 | 62/87 | 7 | 26.3 |
| -3.35 | -0.02 |  |  |  |  |  |  | 1.28 |  |  | 3.7 | 9.1 | -8.2 | 66/87 | 8 | 26.8 |
| -3.25 | -0.02 |  |  |  |  |  |  | 1.27 | -0.01 |  |  | 10.3 | -8.6 | 64/87 | 8 | 27.6 |
| -3.30 | -0.02 |  | -0.0001 |  |  |  |  | 1.40 |  |  |  | 9.9 | -8.5 | 63/87 | 8 | 27.6 |
| -3.26 | -0.02 |  |  |  |  |  |  | 1.25 |  | 0.00 |  | 10.1 | -8.6 | 64/87 | 8 | 27.6 |
| -2.93 |  |  |  |  | -0.07 |  |  | 1.41 |  |  | 3.5 | 9.3 | -8.3 | 63/86 | 8 | 28.1 |
| -2.83 |  |  |  |  | -0.07 |  |  | 1.42 | -0.02 |  |  | 10.6 | -8.8 | 62/86 | 8 | 28.5 |
| -2.86 |  |  |  |  | -0.07 |  |  | 1.39 |  | 0.00 |  | 10.2 | -8.7 | 61/87 | 8 | 28.7 |
|  |  |  |  |  |  |  |  |  |  |  |  |  |  |  |  |  |
| Response: WC_200_ | |  |  |  |  |  |  |  |  |  |  |  |  |  |  |  |
| 3.69 |  |  |  |  | 0.32 |  |  |  |  |  |  |  | 33.9 | 19/64 | 5 | 336.1 |
| 6.16 | 0.10 |  |  |  |  |  |  |  |  |  |  |  | 33.6 | 17/64 | 5 | 337.2 |
| 4.94 |  | 0.20 |  |  |  |  |  |  |  |  |  |  | 35.6 | 12/64 | 5 | 338.1 |
| 3.34 |  |  |  |  | 0.33 |  |  |  |  |  | 11.9 |  | 33.4 | 19/64 | 6 | 338.4 |
| 4.14 |  |  |  |  | 0.32 |  | -0.405 |  |  |  |  |  | 34.2 | 19/65 | 6 | 338.4 |
| 7.16 |  |  |  |  |  |  |  |  |  |  |  |  | 34.6 | 5/63 | 4 | 338.4 |
| 3.57 |  |  |  |  | 0.32 |  |  |  | 0.05 |  |  |  | 33.4 | 19/64 | 6 | 338.4 |
| 3.57 |  |  |  |  | 0.33 |  |  |  |  |  |  | 5.1 | 30.9 | 19/64 | 6 | 338.5 |
| 3.62 |  |  |  |  | 0.32 |  |  |  |  | 0.02 |  |  | 34.1 | 19/65 | 6 | 338.5 |
| 3.66 |  |  |  |  | 0.32 |  |  | 0.02 |  |  |  |  | 34.1 | 19/65 | 6 | 338.5 |
| 4.05 |  |  |  |  | 0.38 |  |  |  |  |  |  | 28.3 |  | 18/62 | 5 | 338.8 |
| 5.94 | 0.09 |  | 0.0005 |  |  |  |  |  |  |  |  |  | 34.2 | 18/66 | 6 | 338.9 |
| 6.61 |  |  | 0.0008 |  |  |  |  |  |  |  |  |  | 35.2 | 9/64 | 5 | 339.2 |
| 5.71 |  |  |  |  | 0.32 |  |  |  |  |  |  |  |  | 16/58 | 4 | 339.4 |
| 5.82 | 0.10 |  |  |  |  |  |  | 0.72 |  |  |  |  | 34.0 | 17/66 | 6 | 339.4 |
| 5.92 | 0.11 |  |  |  |  |  |  |  |  |  | 10.5 |  | 33.1 | 17/65 | 6 | 339.5 |
| 6.12 | 0.11 |  |  |  |  |  |  |  |  |  |  | 4.5 | 30.9 | 17/64 | 6 | 339.5 |
| 6.12 | 0.11 |  |  |  |  |  |  |  | 0.02 |  |  |  | 33.4 | 17/64 | 6 | 339.6 |
| 6.07 | 0.10 |  |  |  |  |  |  |  |  | 0.03 |  |  | 33.8 | 16/66 | 6 | 339.6 |
| 6.18 | 0.10 |  |  |  |  |  | -0.023 |  |  |  |  |  | 33.9 | 16/66 | 6 | 339.6 |
| 6.91 | 0.13 |  |  |  |  |  |  |  |  |  |  | 27.9 |  | 16/63 | 5 | 339.8 |
| 5.05 |  | 0.16 | 0.0004 |  |  |  |  |  |  |  |  |  | 35.8 | 13/65 | 6 | 340.1 |

| **Supplementary material** Table S2. The ranges and means of the traits measured for 13 *Sphagnum* species. The structural traits numerical density (ND), capitulum mass area (CMA) and bulk density (BD) and the height above the water table (HWT) are measured at sample level, N=73, 4-5 per species, while the morphological and anatomical traits were measured at species level, N=15, 1 per species. | | |
| --- | --- | --- |
| Trait | Range | Mean |
| ND (cm^-2^) | 0.82-13.37 | 3.10 |
| CMA (g cm^-2^) | 0.43-6.59 | 2.52 |
| BD 1 (g cm^-3^) | 0.004-0.05 | 0.02 |
| BD 2 (g cm^-3^) | 0.009-0.11 | 0.04 |
| BD 3 (g cm^-3^) | 0.03-0.13 | 0.06 |
| Leaf length (mm) | 0.26-1.95 | 1.19 |
| Leaf width (mm) | 0.66-1.21 | 0.46 |
| Dorsal pore number | 0-3576 | 631.4 |
| Dorsal pore area (%) | 0-29.21 | 9.77 |
| Dorsal pore size (µm) | 0-17.63 | 10.72 |
| Dorsal chlorophyll exposure (%) | 0-0.299 | 0.11 |
| Ventral pore number | 18-1100 | 303.5 |
| Ventral pore area (%) | 0.22-14.62 | 3.93 |
| Ventral pore size (µm) | 5.93-18.62 | 10.75 |
| Ventral chlorophyll exposure (%) | 0-0.31 | 0.09 |
| HWT (mm) | 7-647 | 230.4 |

| **Supplementary material** Table S3. Species-specific asymptotic regression models were fitted for the relationship between F_v_/F_m_ and water content. Pseudo-R^2^ was calculated based on the residuals (Lefcheck, 2016): 𝜎_fitted_/(𝜎_fitted_  + 𝜎_residuals_). | | | |
| --- | --- | --- | --- |
|  |  |  |  |
| *Sphagnum* species | | Pseudo-R^2^ |  |
| *S. angustifolium* | | 0.18 |  |
| *S. balticum* | | 0.52 |  |
| *S. cuspidatum* | | 0.74 |  |
| *S. fuscum* bog | | 0.22 |  |
| *S. fuscum* fen | | 0.12 |  |
| *S. fallax* | | 0.93 |  |
| *S. girgensohnii* | | 0.37 |  |
| *S. lindbergii* | | 0.23 |  |
| *S. magellanicum* open bog | | 0.43 |  |
| *S. magellanicum* pine bog | | 0.23 |  |
| *S. majus* | | 0.85 |  |
| *S. papillosum* | | 0.39 |  |
| *S. rubellum* | | 0.20 |  |
| *S. tenellum* | | 0.92 |  |
| *S. warnstorfii* | | 0.34 |  |
|  |  | |  |

**Lefcheck JS.** 2016 piecewiseSEM: Piecewise structural equation modeling in R for ecology, evolution, and systematics. Methods in Ecology and Evolution. **7**(5): 573-579. doi: 10.1111/2041-210X.12512


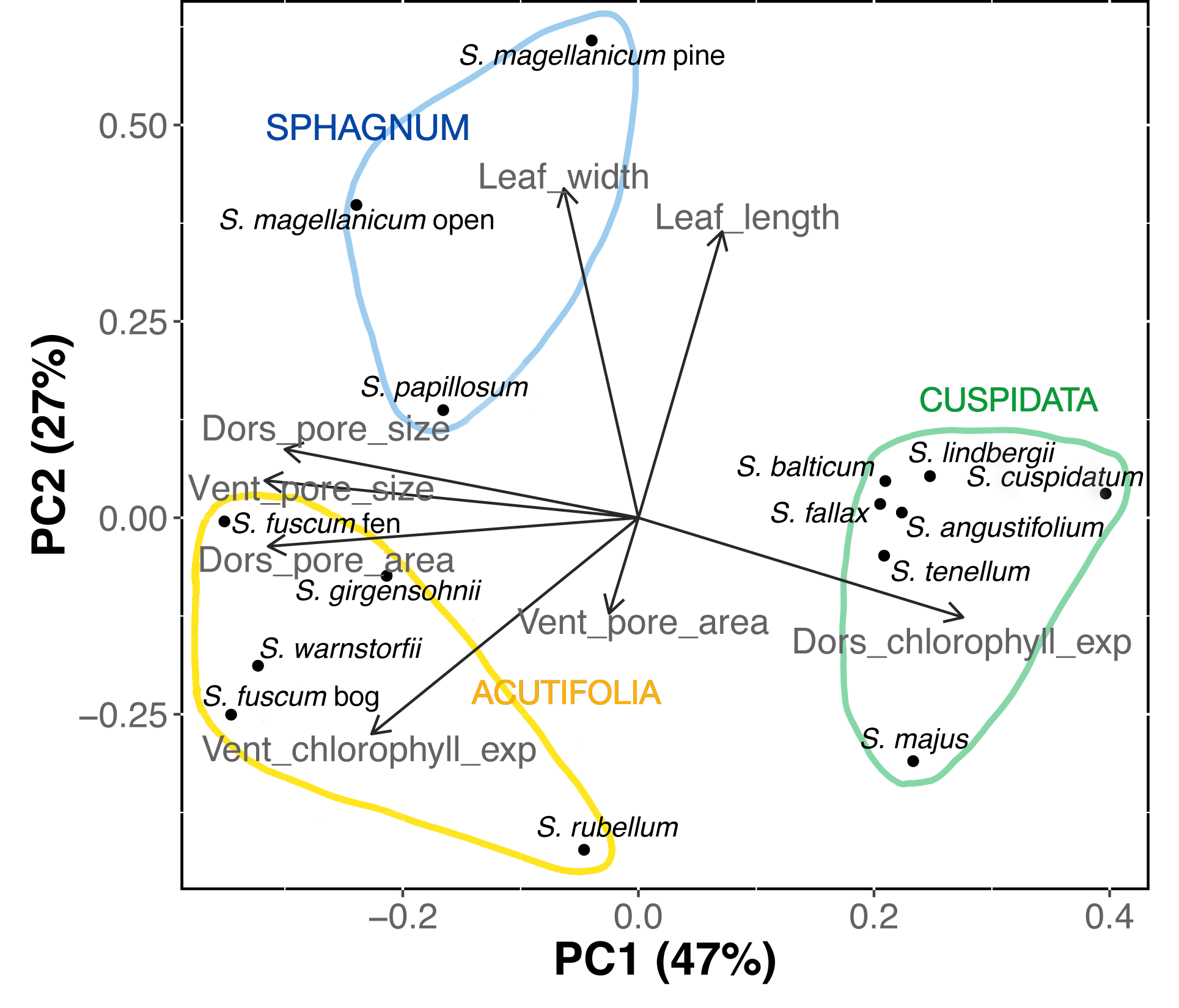


**Supplementary material** Figure S1. PCA of the leaf traits of the *Sphagnum* species (including samples of *S. fuscum* and *S. magellanicum* from two habitats each). Leaf_length and Leaf_width: length and width of leaf (μm). Dors_pore_size and Vent_pore_size: diameter (μm) of pores exposed on the dorsal and ventral leaf side, respectively. Dors_pore_area and Vent_pore_area: pore area as percentage of leaf area, on dorsal and ventral leaf sides, respectively. Dors_chlorophyll_exp and Vent_chlorophyll_exp: proportion of transverse section of leaf that is chlorophyll cell, on dorsal and ventral leaf sides, respectively.
